# Assessing the effect of HAART on change in quality of life among HIV-infected women

**DOI:** 10.1186/1742-6405-3-6

**Published:** 2006-03-20

**Authors:** Chenglong Liu, Kathleen Weber, Esther Robison, Zheng Hu, Lisa P Jacobson, Stephen J Gange

**Affiliations:** 1 Department of Epidemiology, Johns Hopkins Bloomberg School of Public Health, Baltimore, MD, USA; 2 Department of Medicine, Georgetown University School of Medicine, Washington, DC, USA; 3 The CORE Center at John H. Stroger Jr Hospital of Cook County, Chicago, IL, USA; 4 Montefiore Medical Center, New York, NY, USA

## Abstract

**Background:**

The impact of highly active antiretroviral therapy (HAART) on health-related quality of life (QOL) of HIV-1 infected individuals in large prospective cohorts has not been well studied.

**Objective:**

To assess the effect of HAART on QOL by comparing HIV-infected women using HAART with HIV-infected women remaining HAART naïve in the Women's Interagency HIV Study (WIHS), a multicenter prospective cohort study begun in 1994 in the US.

**Methods:**

A 1:1 matching with equivalent (≤ 0.1%) propensity scores for predicting HAART initiation was implemented and 458 pairs were obtained. HAART effects were assessed using pattern mixture models. The changes of nine QOL domain scores and one summary score derived from a shortened version of the MOS-HIV from initial values were used as study outcomes.

**Results:**

The background covariates of the treatment groups were well-balanced after propensity score matching. The 916 matched subjects had a mean age of 38.5 years and 42% had a history of AIDS diagnosis. The participants contributed a total of 4,292 person visits with a median follow-up time of 4 years. In the bivariate analyses with only HAART use and time as covariates, HAART was associated with short-term improvements of 4 QOL domains: role functioning, social functioning, pain and perceived health index. After adjusting for demographic, socioeconomic, biological and clinical variables, HAART had small but significant short-term improvements on changes in summary QOL (mean change: 3.25; *P *= 0.02), role functioning (6.99; *P *< 0.01), social functioning (5.74; *P *< 0.01), cognitive functioning (3.59; *P *= 0.03), pain (6.73; *P *< 0.01), health perception (3.67; *P *= 0.03) and perceived health index (4.87; *P *< 0.01). These QOL scores typically remained stable or declined over additional follow-up and there was no indication that HAART modified these trends.

**Conclusion:**

Our study demonstrated significant short-term HAART effects on most QOL domains, but additional use of HAART did not modify long-term trends. These changes could be attributed to the direct effect of HAART and indirect HAART effect mediated through clinical changes.

## Background

As an important measure of self-reported health and well-being, health-related quality of life (QOL) has been widely applied in evaluating treatment effects among different populations[1]. The effectiveness of highly active antiretroviral therapy (HAART) in arresting viral replication and reducing HIV-related morbidity and mortality has been consistently demonstrated [2-4]; however, its impact on QOL has been unclear.

Published study findings have varied between reporting positive[5,6] or negative effects of HAART on QOL[7,8], with documented improvements often of minimal or modest change [9-12]. A number of these studies have been nested in clinical trials which are typically of short duration and enroll selected study populations[13,14] resulting in under-representation of women, minorities, and substance users who now comprise an increasingly important demographic component of the HIV epidemic[14,15], Observational studies offer an opportunity to examine long-term changes in more heterogeneous populations. However, without randomized treatment assignments, these studies may be influenced by unbalanced distributions of disease stage and background covariates that complicate unconfounded comparisons of treatment groups[16]. Although HAART has been available since the introduction of protease inhibitors in 1996, its long-term effect on QOL has rarely been assessed in large prospective cohort studies[17].

The primary objective of this study was to assess the effect of HAART on QOL change by comparing HIV-infected women using HAART with women remaining HAART naïve. To evaluate this question, we utilized data from the Women's Interagency HIV Study (WIHS), one of the largest prospective cohort studies of HIV-infected and at-risk women in the U.S. Acknowledging the challenges encountered in the analysis of observational data, we utilized methods that balanced the distributions of many background covariates through matching based upon a propensity score, the estimated probability of HAART initiation, and effectively handled informative drop-out by using a pattern mixture model.

## Methods

### Study population

The WIHS is a multicenter prospective study designed to explore the natural and treated history of HIV disease among women since 1994. The WIHS study design and methods are detailed elsewhere[18]. Briefly, a total of 3,768 HIV-seropositive or high risk HIV-negative women aged 13 years or older were recruited from six consortia sites located in Chicago, Los Angeles, San Francisco, Washington D.C., Brooklyn and Bronx in New York City. The study was approved by the local institutional review board at each site and informed consent was obtained for all participants. Research visits are conducted semiannually and include extensive questionnaire-based interviews, specimen collection, physical and obstetric/gynecologic examination. Self-reported quality of life was ascertained at each semiannual visit through 1999 and annually thereafter. This analysis uses data collected through September 2004 (study visit 20). For this study, a matched cohort design was adopted and our analyses were restricted to the HIV-positive participants who enrolled in WIHS during 1994–1995 and had at least one QOL measurement after the matching (baseline) visit as described in detail below.

### Study variables

Among many QOL instruments used for HIV-infected populations, the Medical Outcome Study (MOS)-HIV has been one of the most widely used disease specific instruments. In WIHS, a shortened version of MOS-HIV developed by Bozzette et al[19] was adopted to measure QOL. With this instrument, item redundancy is reduced while excellent reliability is maintained and construct validity is comparable to that of MOS-HIV. The shortened form has 21 items representing 9 domains: physical functioning, role functioning, energy/fatigue, social functioning, cognitive functioning, pain, emotional well-being, perceived health index and current health perception. The domain scores are derived by averaging the recoded raw scores for corresponding items of each domain expressed on a 0–100 scale, with higher values for better functioning and well-being according to an established scoring recommendation. In addition, one summary score is generated from six domains (physical functioning, role functioning, energy/fatigue, social functioning, pain and emotional well-being) on the basis of a published algorithm[19]. The summary and nine domain scores are the outcomes of interest in this study.

HAART was defined following the Department of Health and Human Service/Kaiser Panel guidelines[20] and defined as: (a) two or more nucleoside reverse transcriptase inhibitors (NRTIs) in combination with at least one protease inhibitor (PI) or one non-nucleoside reverse transcriptase inhibitor (NNRTI); (b) one NRTI in combination with at least one PI and at least one NNRTI; and (c) an abacavir or tenofovir containing regimen of three or more NRTIs in the absence of both PIs and NNRTIs. Combinations of zidovudine (AZT) and stavudine (d4T) with either a PI or NNRTI were not considered HAART. While HAART use can vary over time, in this analysis we consider trends following first HAART initiation.

On the basis of results from prior studies and data available in WIHS, we selected a number of variables possibly affecting participants' and/or provider's decision to initiate HAART or their QOL. Age was determined at the matching visit. Race/ethnicity was categorized as White non-Hispanic, Black non-Hispanic, Latina/Hispanic and other. Education level at study entry was coded as less than high school, completed high school, and above high school. Annual gross income was dichotomized as greater than $12,000 or not. The number of HIV-related constitutional symptoms, including fever, diarrhea, memory problems, neuropathy symptoms (numbness, tingling or burning), unintentional weight loss, confusion and night sweats, were aggregated for each visit. Standardized three or four color flow cytometry was used to determine total CD4+ cells/mm^3 ^at laboratories concurrently[21] at each visit. Plasma HIV-1 RNA levels were measured using the isothermal nucleic acid sequence based amplification (NASBA/Nuclisens) method (bioMérieux, Boxtel, NL) in laboratories participating in the NIH/NIAID Virology Quality Assurance Laboratory proficiency testing program. The current lower limit of quantification was 80 copies/ml using 1.0 ml sample input. Self-reported depressive symptoms was measured using the 20-item Center for Epidemiological Studies Depression Scale (CES-D)[22], with a total score of 16 or greater used to define the presence of depression. Current employment, any insurance coverage, clinical AIDS diagnosis, and the number of outpatient visits, hospitalizations and medications taken (antiretroviral and non-antiretroviral) since last visit, were also included in our analysis. As calendar time affected the chance of HAART initiation[3,16], it was also included as a covariate in estimating propensity score.

### Statistical analysis

#### Propensity score matching

Unlike in randomized trials, use of therapies in observational studies is not from random assignment and thus unbalanced distributions of background confounders may bias the estimated exposure effects. To account for this, conventional matching or stratification methods can sometimes be used to create groups of exposed and unexposed individuals with similar measured covariates. Given the large number of background covariates and limited sample size in most observational studies, it is often implausible to control all covariates at one time in this way. As an alternative, propensity score methods have been developed[23] that attempt to match or stratify on a scalar propensity score that reflects an individual's estimated probability of taking a treatment conditional on other variables. By selecting exposed and unexposed individuals matched on the propensity score, we eliminate the associations between HAART initiation and these covariates; thus, these factors will not serve as confounders when we evaluate the effect of HAART. As many factors could affect HAART initiation in WIHS, it is reasonable to use propensity score matching to help eliminate indication bias.

To construct the propensity score of initiating HAART in our analysis, a multiple logistic regression method was used. For the HAART users, we selected the last visit before HAART initiation as the matching visits. For the HAART naïve HIV positive women, we included all of their QOL visits as candidate matching visits. The matching visit data from the HAART exposed group and the candidate matching visit data from the HAART naïve group were pooled together and a propensity score was obtained for each participant at each visit conditional on a number of variables, including age, education, race/ethnicity, income, employment, health insurance, CD4+ cell counts, viral load, history of AIDS diagnosis, clinical depression, and number of symptoms, outpatient visits, hospitalizations and medications, QOL scores and calendar time. Every HAART user was matched to one randomly selected HAART naïve participant at a baseline visit with an equivalent (within 0.1% rounding level) propensity score of HAART initiation. For any HAART unexposed individual selected as a control, the rest of her visits were removed to ensure 1:1 matching. To evaluate the effect of propensity score matching, T tests and chi-square tests were performed to test differences in the distributions of background variables between the exposed and unexposed groups before and after matching.

#### Pattern mixture model analysis

After matching, the differences of the QOL summary score and the nine domain scores at each visit from their values at the matching baseline visit were used as the study outcomes. To evaluate the effect of HAART, a conventional random effects mixture model could be fit if data were missing only at random, e.g. not related to study outcomes. However, in our analysis, a substantial proportion (33%) of participants, especially those from the HAART naïve group (46%), died during the study follow-up. To obtain a better estimate of changes over time, we utilized a pattern mixture model approach where data were stratified by the pattern of follow-up and distinct models were constructed within each stratum[24] To implement this approach, we grouped the drop-out times into 4 categories (≤ 2, 2.1–4, 4.1–6, and ≥ 6 years) and assumed that the distribution of response would be a weighted mixture over drop-out categories[25]. The overall estimates of variable coefficients and standard errors were obtained across the pattern.

In each model, we included an overall intercept term, a binary indicator for HAART vs. HAART-naïve groups, and a variable reflecting the time (in per 6 months) from the baseline visit, which formed Model 1. Thus, the HAART indicator reflects short-term effects of HAART and the term for time reflects whether this change persists over follow-up. To assess if HAART impacts the overall long-term trend, we fit interaction terms between HAART and time. Furthermore, in order to account for residual confounding and explore possible mediators of how HAART exerted its effect on QOL, a series of models were fit with different combinations of covariates added to previous models: Model 2 added baseline age, ethnicity, and education variables to Model 1, Model 3 added time-varying socioeconomic variables of income, employment, and health insurance to Model 2, Model 4 added time-varying CD4+ cell counts and viral load to Model 3, and Model 5 added time varying symptoms, outpatient visits, hospitalizations, medications, AIDS and depression to Model 4. All statistical analyses were performed using a SAS version 9.1 (SAS Institute, Cary, NC) and Splus 7.0 (Insightful, Seattle, WA).

## Results

**Figure 1:**
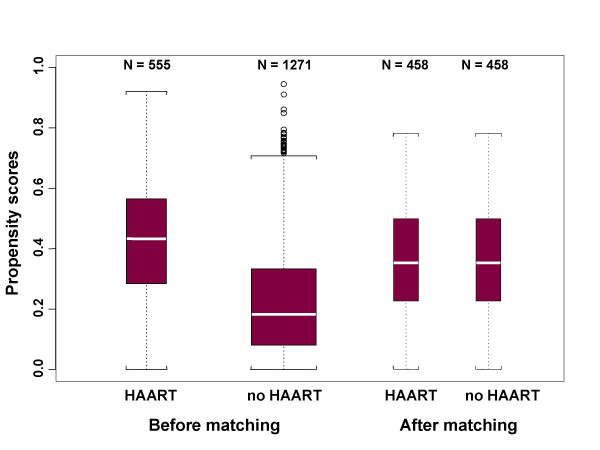
Boxplots of QOL summary score between HAART users and HAART naïve groups before and after propensity-score matching. Box widths are proportional to the number of observations in each group.

Table 1 displays the differences in the distributions of baseline covariates between the HAART users and HAART-naïve groups before and after matching. Prior to propensity score matching, the distributions of risk factors affecting HAART initiation were compared between 1,271 HAART exposed (the last visits before matching) and 555 HAART naïve participants (at candidate matching visits). Thirteen out of the 24 background covariates, including education level, race/ethnicity, income, insurance, CD4+ cell counts, viral load, AIDS diagnosis, number of symptoms, outpatient visits and medications, physical functioning, perceived health index and health rating, were significantly different between the groups, which necessitated the matching of these covariates in our study. Using a tolerance of 0.1% in the propensity score, we were able to obtain 458 matched pairs of HAART initiators and HAART naïve women. No statistically significant differences were observed for any of these background covariates after matching (Table 1), which demonstrated a success in matching the covariates as expected. The resulting distributions of propensity scores for the two groups before and after matching are displayed in Figure 1. Before matching, the average propensity scores for HAART using and naïve groups were 0.42 and 0.22 respectively. However, after propensity score matching, the distributions of propensity scores were nearly identical (mean: 0.36; standard deviation: 0.17 for both groups).

**Table 1:** Study Participant Characteristics Before and After Propensity Score Matching. Numbers indicate mean value unless otherwise noted.

	**Before Matching**			**After Matching**		
		
**Covariates**	**HAART Naive (N = 555)**	**HAART Users (N = 1271)**	**P-Value**	**HAART Naive (N = 458)**	**HAART Users (N = 458)**	**P-Value**
**Age at baseline**	38.6	38.4	0.42	38.6	38.4	0.75
**Education %**			**<.01**			0.30
**Less than high school**	39.8	35.5		40.8	39.1	
**Completed high school**	33.2	31.2		31.7	29.5	
**College and above**	27.0	33.4		27.5	31.4	
**Race %**			**0.02**			0.35
**White non-Hispanic**	15.1	18.5		16.4	17.5	
**Black non-Hispanic**	62.5	53.8		60.7	56.6	
**Hispanic**	3.9	3.5		4.1	3.1	
**Others**	18.5	24.2		18.8	22.9	
**Annual gross income (>$12,000) %**	35.0	38.9	**0.02**	36.9	37.6	0.84
**Employment %**	26.6	25.3	0.37	21.6	25.5	0.16
**Insurance %**	40.2	23.4	**<.01**	26.6	30.8	0.17
**CD4+ cell count**	533.8	301.9	**<.01**	339.4	344.9	0.74
**Viral Load (log10)**	3.7	4.0	**<.01**	4.2	4.1	0.17
**AIDS diagnosis %**	36.4	44.3	**<.01**	43.2	41.9	0.69
**Depression %**	50.0	47.4	0.11	49.8	49.8	1.00
**Number of symptoms**	1.3	1.5	**0.03**	1.5	1.5	0.84
**Number of outpatient visit**	3.8	5.9	**<.01**	5.1	4.9	0.71
**Number of hospitalizations**	0.3	0.3	0.16	0.4	0.3	0.43
**Number of medications**	2.8	3.8	**<.01**	3.7	3.4	0.13
**Quality of life scores**						
**Physical functioning**	66.2	64.1	**0.03**	63.1	65.5	0.23
**Role functioning**	74.0	73.0	0.28	74.0	74.1	0.98
**Energy/Fatigue**	54.1	53.0	0.20	51.6	53.1	0.39
**Social functioning**	72.0	72.0	0.92	73.0	72.2	0.65
**Cognitive functioning**	76.7	78.1	0.09	78.6	77.1	0.33
**Pain**	69.9	69.5	0.64	70.2	69.1	0.55
**Emotion well-being**	59.8	59.9	0.95	59.1	59.0	0.94
**Perceived health index**	53.7	51.6	**0.01**	50.8	52.2	0.40
**Health rating**	66.4	68.1	**0.02**	66.9	67.1	0.89
**Summary score**	63.2	62.3	0.19	61.9	62.5	0.65

The 916 matched participants had a mean age of 38.5 years at baseline and contributed a total of 4,292 person visits, with a median follow-up time of 4 years (interquartile range (IQR): 1–6 years). Among these women, about 58% were Black, non-Hispanics, 60% completed high school and 42% had an AIDS history at the matching visits. At baseline, the average CD4+ cell count was approximately 340 cells/mm^3^, the mean viral load was approximately 10,000 copies/ml and the mean QOL summary score was 62. About 63% of HAART naïve women dropped during the first two years, while the percentage was only 11% for women using HAART. In contrast, only 11% of HAART naïve women were followed for 6 or more years whereas the percentage for the women using HAART was 38%.

To evaluate how HAART affected QOL change, we fit a series of pattern mixture models with different subsets of covariates (Table 2). In each model, HAART use and time from matching visits were included. We first examined whether there were any significant interactions between time and HAART use to assess any long-term HAART effect on QOL score changes. As the interaction terms were not statistically significant in any model (though its direction was positive), it was dropped out from our analyses. Then, we evaluated the overall effect of HAART on changes of QOL scores (the summary score and nine specific QOL domain scores) without time varying intermediate variables (models 1–2) and the direct effects of HAART after adjusting for different possible mediating covariates (models 3–5).

Compared with the HAART naïve group in the bivariate model (Model 1) with HAART use and time as the only covariates, the HAART users had improved QOL scores from the matching visits for almost all domains except for energy/fatigue, with those for role functioning (mean change: 5.08; *P *= 0.01), social functioning (4.33; *P *= 0.01), pain (4.53; *P *= 0.01) and perceived health index (4.25; *P *< 0.01) reaching a statistically significant level. A second model (Model 2) was fit by adding fixed personal characteristics, including age at baseline, race/ethnicity and education at study enrollment, into the bivariate model. The model estimates for HAART and time changed slightly except for cognitive functioning, which became statistically significant (3.51; *P *= 0.02). In Model 3, we included time-dependent socioeconomic variables – income, employment and health insurance into the Model 2. No significant change of HAART effect was observed. After further adding markers for disease progression (CD4+ cell counts and HIV viral load) as in the Model 4, the HAART effects remained stable except for health perception (3.43; *P *= 0.04). In the final model (Model 5), the clinical variables (number of symptoms, outpatient visits, hospitalizations and medications, history of AIDS diagnosis and clinical depression) were added as covariates into the Model 4. Except for cognitive functioning, health perception and perceived health index, adding clinical variables into the models was associated with biggest changes in HAART effect estimates. In addition, the direct HAART effect on summary QOL change became significant (3.25; *P *= 0.02). Furthermore, though the QOL scores decreased over time for almost all domains in all models, only the decreases of summary QOL, role functioning, emotional well-being and health perception were statistically significant in the final model after controlling many time varying covariates.

As the HIV-infected individuals at different disease stages might have different responses to HAART, we further examined the association of HAART and QOL among women who were AIDS-free at the matching visits (Table 3). Again, all QOL domain scores remained stable or decreased (for health perception) during follow-up, and HAART use did not modify these trends. Compared to the Table 2, fewer QOL domains were significant for short-term HAART effects (social function, pain and health rating) and it was negative for the energy/fatigue domain.

In addition to HAART use and time, a number of the covariates were significantly associated with QOL changes from baseline. Evaluating the results from Model 5 for the summary QOL change, women having less than high school education had slightly higher summary QOL change (3.12; *P *= 0.02) compared to women with college education at study enrollment. In addition, all clinical variables were significantly associated with summary QOL change. Having one more symptom, outpatient visit, hospitalization or medication was associated with a decrease of 2.17 (*P *< 0.01), 0.11 (*P *< 0.02), 1.57 (*P *< 0.01) or 0.24 (*P *< 0.01) in summary QOL change respectively. Depression was strongly related to a decline in summary QOL change (-9.78; *P *< 0.01), while having a history of clinical AIDS was associated with improved QOL change (2.13; *P *= 0.04). All other demographic, socioeconomic and biological (CD4+ cell counts and HIV viral load) variables were not significantly associated with QOL changes from baseline.

**Table 2:** Estimates of the Impact of HAART on the Mean Change in QOL Scores from Propensity-Score Matched Pattern Mixture Models.

		**Model 1**^**a**^		**Model 2**^**b**^		**Model 3**^**c**^		**Model 4**^**d**^		**Model 5**^**e**^	
		**Effect**	**P-Value**	**Effect**	**P-Value**	**Effect**	**P-Value**	**Effect**	**P-Value**	**Effect**	**P-Value**

**Summary QOL Score**	Short-Term HAART Effect	2.07	0.11	2.25	0.07	2.26	0.07	2.24	0.08	3.25	**0.02**
	Change per 6 months	-0.30	**0.01**	-0.30	**0.01**	-0.21	0.08	-0.29	**0.02**	-0.29	**0.01**

**Physical functioning**	Short-Term HAART Effect	0.71	0.70	0.72	0.70	0.83	0.66	0.15	0.94	1.86	0.35
	Change per 6 months	-0.39	**0.02**	-0.39	**0.02**	-0.28	0.14	-0.36	0.07	-0.30	0.10

**Role functioning**	Short-Term HAART Effect	5.08	**0.01**	5.40	**0.01**	5.46	**0.01**	5.98	**0.01**	6.99	**0.00**
	Change per 6 months	-0.40	**0.04**	-0.36	0.07	-0.34	0.11	-0.47	**0.03**	-0.45	**0.04**

**Energy/fatigue**	Short-Term HAART Effect	-0.09	0.96	0.37	0.83	0.32	0.86	0.43	0.81	1.24	0.51
	Change per 6 months	-0.30	0.06	-0.31	0.05	-0.19	0.28	-0.21	0.24	-0.26	0.13

**Social functioning**	Short-Term HAART Effect	4.33	**0.01**	4.96	**0.00**	4.90	**0.01**	5.03	**0.01**	5.74	**0.00**
	Change per 6 months	-0.22	0.19	-0.19	0.25	-0.13	0.47	-0.19	0.30	-0.19	0.30

**Cognitive functioning**	Short-Term HAART Effect	2.58	0.08	3.51	**0.02**	3.46	**0.02**	3.48	**0.03**	3.59	**0.03**
	Change per 6 months	-0.08	0.57	-0.07	0.61	0.07	0.64	0.04	0.78	-0.04	0.82

**Pain**	Short-Term HAART Effect	4.53	**0.01**	4.33	**0.02**	4.33	**0.02**	4.65	**0.02**	6.73	**0.00**
	Change per 6 months	-0.25	0.15	-0.26	0.14	-0.10	0.58	-0.21	0.29	-0.19	0.32

**Emotional well-being**	Short-Term HAART Effect	2.38	0.12	2.46	0.12	2.47	0.12	2.45	0.12	2.05	0.20
	Change per 6 months	-0.16	0.25	-0.17	0.23	-0.18	0.23	-0.26	0.11	-0.30	**0.05**

**Health perception**	Short-Term HAART Effect	2.60	0.10	3.05	0.06	3.04	0.06	3.43	**0.04**	3.67	**0.03**
	Change per 6 months	-0.49	**0.00**	-0.49	**0.00**	-0.45	**0.00**	-0.50	**0.00**	-0.53	**0.00**

**Perceived health index**	Short-Term HAART Effect	4.25	**0.00**	4.79	**0.00**	4.67	**0.00**	4.65	**0.00**	4.87	**0.00**
	Change per 6 months	-0.08	0.86	-0.09	0.87	-0.11	0.87	-0.11	0.84	-0.09	0.83

^**a **^Model 1: Model includes an intercept, an indicator of HAART or HAART-naïve group (*Short-Term HAART Effect*) and the time from index visit (*Change per 6 months*).

^**b **^Model 2: Model 1 + age, ethnicity and education level.

^**c **^Model 3: Model 2 + income, employment and health insurance.

^**d **^Model 4: Model 3+ CD4+ cell counts and viral load.

^**e **^Model 5: Model 4+ number of symptoms, outpatient visits, hospitalizations and medication, AIDS history and clinical depression. Interactions between HAART and time from index visit were not statistically significant for all models.

**Table 3:** Estimates of the Impact of HAART on the Mean Change in QOL Scores from Propensity-Score Matched Pattern Mixture Models among AIDS-free Women at Matching Visits

		**Model 1**^**a**^		**Model 2**^**b**^		**Model 3**^**c**^		**Model 4**^**d**^		**Model 5**^**e**^	
		**Effect**	**P-Value**	**Effect**	**P-Value**	**Effect**	**P-Value**	**Effect**	**P-Value**	**Effect**	**P-Value**

**Overall QOL**	Short-Term HAART Effect	0.57	0.70	0.40	0.79	0.43	0.78	0.63	0.69	1.61	0.35
	Change per 6 months	-0.24	0.10	-0.26	0.08	-0.19	0.23	-0.26	0.11	-0.20	0.16

**Physical functioning**	Short-Term HAART Effect	0.05	0.98	-0.19	0.94	-0.23	0.92	-0.88	0.73	0.55	0.83
	Change per 6 months	-0.27	0.25	-0.28	0.23	-0.26	0.31	-0.37	0.14	-0.25	0.26

**Role functioning**	Short-Term HAART Effect	2.48	0.33	2.61	0.30	2.73	0.28	3.03	0.24	4.13	0.12
	Change per 6 months	-0.30	0.25	-0.26	0.32	-0.28	0.33	-0.33	0.26	-0.26	0.35

**Energy/fatigue**	Short-Term HAART Effect	-2.22	0.30	-2.12	0.31	-2.14	0.31	-1.67	0.44	-0.71	0.75
	Change per 6 months	-0.25	0.22	-0.30	0.16	-0.19	0.42	-0.26	0.27	-0.24	0.28

**Social functioning**	Short-Term HAART Effect	3.17	0.12	3.71	0.08	3.85	0.08	4.33	0.05	5.27	**0.02**
	Change per 6 months	-0.17	0.43	-0.16	0.44	-0.08	0.73	-0.09	0.71	0.01	0.97

**Cognitive functioning**	Short-Term HAART Effect	0.86	0.61	2.21	0.22	2.23	0.22	2.54	0.16	2.38	0.22
	Change per 6 months	-0.13	0.46	-0.11	0.52	-0.05	0.79	-0.06	0.76	-0.11	0.54

**Pain**	Short-Term HAART Effect	3.25	0.13	2.53	0.24	2.69	0.21	3.46	0.13	5.96	**0.02**
	Change per 6 months	-0.21	0.36	-0.26	0.28	-0.11	0.67	-0.17	0.52	-0.11	0.66

**Emotional well-being**	Short-Term HAART Effect	1.56	0.40	1.06	0.58	1.06	0.58	1.02	0.58	0.17	0.93
	Change per 6 months	-0.19	0.32	-0.18	0.34	-0.16	0.42	-0.20	0.36	-0.23	0.26

**Health perception**	Short-Term HAART Effect	0.07	0.97	0.36	0.84	0.27	0.88	0.31	0.87	0.45	0.82
	Change per 6 months	-0.36	**0.04**	-0.37	**0.04**	-0.37	0.06	-0.50	**0.01**	-0.54	**0.00**

**Perceived health index**	Short-Term HAART Effect	3.17	0.07	3.76	**0.03**	3.46	0.05	4.03	**0.02**	4.08	**0.03**
	Change per 6 months	-0.21	0.21	-0.20	0.22	-0.28	0.11	-0.31	0.08	-0.26	0.14

^**a **^Model 1: Model includes an intercept, an indicator of HAART or HAART-naïve group (*Short-Term HAART Effect*) and the time from index visit (*Change per 6 months*).

^**b **^Model 2: Model 1 + age, ethnicity and education level.

^**c **^Model 3: Model 2 + income, employment and health insurance.

^**d **^Model 4: Model 3+ CD4+ cell counts and viral load.

^**e **^Model 5: Model 4+ number of symptoms, outpatient visits, hospitalizations and medication, AIDS history and clinical depression. Interactions between HAART and time from index visit were not statistically significant for all models.

## Discussion

In our study, we attempted to obtain unbiased estimates of HAART effects on QOL in WIHS by minimizing indication bias and further adjusting for the effect of informative drop-outs using several innovative statistical methods. By balancing the distributions of observed background covariates using propensity score matching, the observational studies come closer to mimicking the effect of randomized clinical trials with equivalent probability of receiving treatment. In addition, application of joint modeling skills like pattern mixture model is one method to handle the informative drop-outs which may bias effect estimates in longitudinal studies.

Our study showed that HAART improved most QOL domains relatively quickly. Most of these domains were stable or showed slight declines over subsequent follow-up, and there was no indication that HAART modified these trends. These results suggest that continued use of HAART did not result in continued improvement in QOL domains. This lack of long-term effect might reflect a balance between reduced HIV-related symptoms and added side effects from HAART. As many time-dependent variables were controlled already, the likely explanation for QOL decrease over time might be due to aging or other uncontrolled factors. It should be noted that the QOL decrease trends were not entirely homogeneous. Examining results from different drop-out patterns revealed that women with the shortest maximum follow-up time had the highest rate of QOL decrease in both groups (data not shown). As early drop-out due to causes like death is usually associated with faster disease progression and quicker deterioration of QOL, appropriate handling of informative drop-outs using a pattern mixture model was justified in our analysis.

By adding different combinations of covariates step by step into the models, we could explore the possible mediators through which HAART renders its effect. In the bivariate models, HAART use had positive overall effects for almost all QOL domains, which is congruent with some clinical trial results with relative short follow-up periods[10,11]. Because fixed demographic covariates were already controlled at baseline by matching, it is not surprised that adding these variables did not substantially alter the estimated HAART effects. Addition of time varying socioeconomic variables did not change the estimates much either, indicating that these covariates had been stable through the study follow-up. Though HAART could decrease viral load dramatically and increase CD4+ cell counts accordingly, the observed HAART effects did not differ substantially with and without these variables in the models. This phenomenon might be explained by the weak associations between these biological variables and QOL[1,26]. Finally, with the inclusion of the time varying clinical variables, the estimates of HAART effect experienced the biggest improvement for most QOL domains, providing evidence that these clinical covariates served as mediate factors and had negative impacts on QOL. In addition, the significance of direct HAART effects on most QOL domain scores implies that HAART might have rendered its effect through pathways other than improving the patient's immune status or changing clinical profile. One of the multiple possible explanations for this may be simply a placebo effect resulting from relieved stress for the infected individuals[27] using HAART because the effectiveness of HAART in reducing AIDS-related morbidity and mortality has been demonstrated. Similar to previous studies[6,7], HAART had different effects for individuals at different disease stages, with short-term improvements of all QOL domains for AIDS patients and deterioration of certain QOL domain for AIDS-free HIV-infected individuals. Thus, it would be advisable to think about the timing of initiating HAART, especially for those individuals at their early stage of HIV disease, to maximize their quality of life.

The propensity score method has been widely applied in observational studies through matching, stratification, or weighting to obtain estimates that may be less biased, more robust and precise[28]. By generating a propensity score from many risk factors affecting HAART initiation, the overall effect of these factors on starting HAART can be represented by this scalar summary score. Through matching with the propensity score, the associations between these risk factors and HAART initiation are blocked and these covariates no longer act as confounders. Noticeably, the distributions of all covariates that were substantially different before matching became identical after matching, which convincingly showed that the matching did what we expected. Furthermore, the HAART effect estimates were relatively stable across models with different combinations of covariates, indicating indirectly that the matching successfully turned many covariates into non-confounders. However, two possible limitations should also be noted. First, we could not find a sufficiently close match for all individuals. In our dataset, the HAART naïve group was smaller (N = 555) than the HAART initiators (N = 1271). In order to have a 1:1 match, we had to restrict to the smaller group, and could only find a match for 83% of these individuals. This is common in propensity score analyses. Second, although the propensity score adjusting method is very effective in balancing the known confounders across groups, omission of important unobserved confounders might still lead to residual confounding in estimating treatment effect. In our study, we included many possible confounders identified from prior studies in estimating the propensity score and examination of other potential variables such as substance abuse and violence history did not show any difference. Thus, the chance of leaving out important confounders was minimized. Of course, omission of unmeasured confounders is a constant threat to the validity of non-interventional studies as well.

In our intent-to-treat analysis, we assumed that individuals who started HAART would remain on HAART throughout the follow-up. Though some participants may have discontinued HAART for a few visits, our data showed that the HAART users had been on HAART for about 80% of their follow-up visits. We did not take into account the adherence to HAART in our analysis though we have controlled some variables, including age and viral load, that contribute to the lower level of adherence to HAART use[29]. In our analysis, we examined the effects of HAART as a whole, rather than the effects of specific HAART regimens on QOL. As HAART regimens vary from individual to individual and from time to time within the same individual in WIHS, it is nearly impossible to assess the effect of every regimen on QOL change given the numerous number of HAART regimens used. In addition, we did not analyze the effect of HAART-related side effects on QOL due to insufficient data. However, as we controlled for clinical variables which are related to both HAART effectiveness and HAART-related side effects, the heterogeneity of HAART regimen effects could be predicted and effect of drug side effects could be partially controlled. In addition, our study subjects are comprised of women at a relatively advanced stages of disease, thus the observed HAART effects may not be representative of the general HIV-infected population.

In WIHS, a shortened version of MOS-HIV form was used to assess QOL change among the participants. The reliability and construct validity of this instrument have been demonstrated and the burden for both investigators and patients was alleviated due to reduced administration time [19]. Though MOS-HIV form has been frequently used in HIV research since the last decade[30], it has relatively limited application among women, minorities and individuals with lower socioeconomic status[31]. As the largest HIV/AIDS prospective cohort of women in the US, the WIHS represents an ethnically diverse, socioeconomically disadvantaged group with complex risk factors whose QOL status has not been well studied. Thus, our analysis will provide important initial information of QOL change for women in the HAART era.

In summary, we evaluated the effects of HAART on QOL among women in the WIHS. HAART did not show any long-term effect on QOL changes, but had short-term direct effects not mediated through clinical variables.

## Competing interests

The author(s) declare that they have no competing interests.
